# Crystal structure of *catena*-poly[[[di­chlorido­copper(II)]-{μ-*tert*-butyl *N*-methyl-*N*-[4-(6-{[4-(pyridin-2-yl-κ*N*)-1*H*-1,2,3-triazol-1-yl-κ*N* ^3^]meth­yl}-1,3-benzo­thia­zol-2-yl)phen­yl]carbamato}] aceto­nitrile monosolvate]

**DOI:** 10.1107/S2056989018000488

**Published:** 2018-01-12

**Authors:** Alexandre Pocinho, Carine Duhayon, Emmanuel Gras, Christelle Hureau

**Affiliations:** a CNRS LCC, Université de Toulouse, 205 route de Narbonne, F-31077 Toulouse, France

**Keywords:** crystal structure, pyridine–triazole, Alzheimer’s disease, copper(II) complex, hydrogen bonding, C—H⋯π inter­actions, offset π–π inter­actions

## Abstract

In the title coordination polymer, {[CuCl_2_(C_27_H_26_N_6_O_2_S)]·CH_3_CN}_*n*_, the copper(II) ion is fivefold coordinated, with an almost perfect square-pyramidal coordination sphere. In the equatorial plane, it is ligated to a pyridine N atom and an N atom of the triazole unit and to two Cl^−^ ions, while the apical position is occupied by the carbonyl O atom of the *tert*-butyl carbamate group. In the crystal, the polymer chains propagate in the [11-1] direction, with the aceto­nitrile solvent mol­ecules linked to the chain by C—H⋯N hydrogen bonds. The chains are linked by C—H⋯Cl hydrogen bonds forming sheets parallel to the plane (011). The crystal packing is further consolidated by C—H⋯π inter­actions and offset π–π stacking inter­actions [inter­centroid distance = 3.6805 (15) Å], forming a three-dimensional supra­molecular structure.

## Abstract 2

The title coordination polymer was obtained by combining an aqueous solution of copper(II) dichloride with the ligand {*tert*-butyl­meth­yl[4-(6-{[4-(pyridin-2-yl-)1*H*-1,2,3-triazol-1-yl]meth­yl}-1,3-benzo­thia­zol-2-yl)phen­yl]carbamate in aceto­nitrile.

## Chemical context   

Alzheimer’s Disease (AD) is a neurodegenerative disease characterized by aggregation of amyloid peptide and extensive inflammation related to a strong oxidative stress (Cheignon *et al.*, 2018 ▸). Metals are known to play a key role in this oxidative stress and also to be associated with peptide aggregation, at the core of the pathology (Faller *et al.*, 2013 ▸; Viles, 2012 ▸). More specifically, Cu^II^ has been found to form a complex with the amyloid peptide for which aggregation is one of the major hallmarks of AD (Eury *et al.*, 2011 ▸; Faller *et al.*, 2014 ▸). This has triggered significant ongoing inter­est in the development of chelators able to inter­act with metals in the context of AD (Santos *et al.*, 2016 ▸; Conte-Daban *et al.*, 2017 ▸).

In the course of our studies on the development of bifunctional mol­ecules able to target amyloid fibrils, for example *via* a 2-aryl­benzo­thia­zole core (Noel *et al.*, 2013 ▸), and inter­act with copper ions found within the senile plaques, we have designed and synthesized a benzo­thia­zole moiety decorated with a triazole-pyridine subunit, *viz. tert*-butyl meth­yl[4-(6-{[4-(pyridin-2-yl)-1*H*-1,2,3-triazol-1-yl]meth­yl} benzo[*d*]thia­zol-2-yl]phen­yl}carbamate (**L**). Indeed integrating the N-binding from the triazole moiety in the binding site of a chelator has been shown to be a successful approach (Jones *et al.*, 2012 ▸, 2017 ▸). Compared to these seminal works, the additional aryl-benzo­thia­zole moiety in compound **L** is expected to enhance the ability of the chelator to inter­act with amyloid aggregates and thus to retrieve deleterious Cu^II^ ions from Aβ fibrils. Investigation of the ability to chelate Cu^II^ ions, by studying the reaction of **L** with CuCl_2_, led to the formation of the title coordination polymer whose synthesis and mol­ecular and crystal structures are described herein.
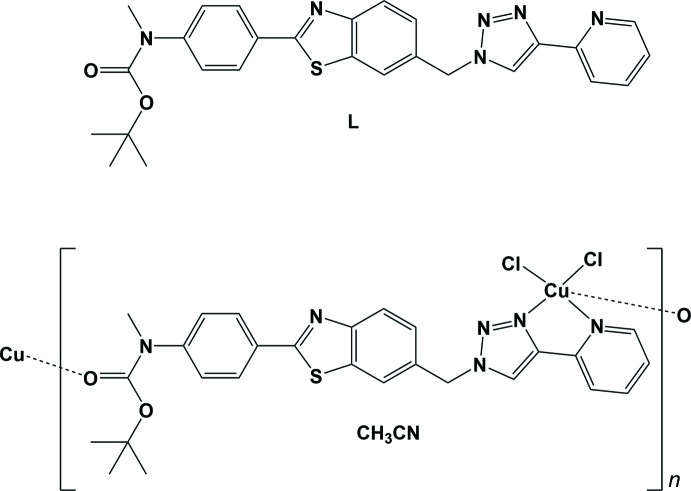


## Structural commentary   

**Figure 1:**
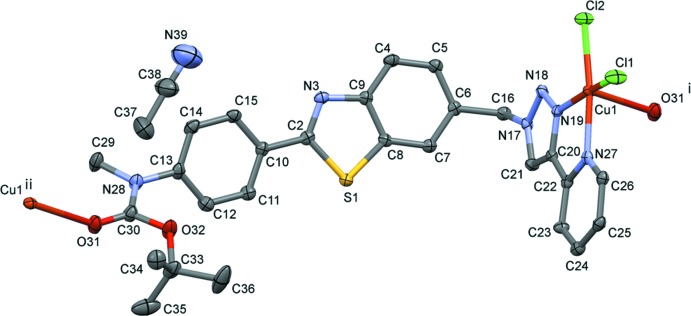
The mol­ecular structure of the asymmetric unit of the title coordination polymer, with atom labelling. Displacement ellipsoids are drawn at the 50% probability level. The H atoms have been omitted for clarity. [Symmetry codes: (i) *x* − 1, *y* − 1, *z* + 1; (ii) *x* + 1, *y* + 1, *z* − 1.]

The mol­ecular structure of the asymmetric unit of the title coordination polymer is shown in Fig. 1 ▸. Selected bond lengths and bond angles are given in Table 1 ▸. The ligand is L-shaped with the benzo­thia­zole ring system (S1/N3/C2/C4–C9; r.m.s. deviation = 0.01 Å) being inclined to the triazole ring (N17-N197C20/C21) by 79.54 (12)°. The benzene ring is inclined to the benzo­thia­zole ring system by 12.27 (11)°, while the pyridine ring is inclined to the triazole ring by 4.07 (14)°. The copper(II) ion is fivefold coordinate with an almost perfect square-pyramidal coordination sphere. In the equatorial plane, the copper(II) ion coordinates the pyridine N atom N27 and atom N19 of the triazole unit and two Cl^−^ anions, while the apical position is occupied by the carbonyl O atom, O31, of the *tert*-butyl­oxycarbamate group. The τ_5_ descriptor for the fivefold coordination sphere is 0.08 (τ_5_ = 0 for an ideal square-pyramidal coordination sphere, and = 1 for an ideal trigonal–pyramidal coordination sphere; Addison *et al.*, 1984 ▸). The triazole ring (N17–N19/C20/C21) exhibits a slightly shorter Cu1—N19 bond length [2.004 (2) Å] than the pyridine Cu1—-N27 bond length [2.054 (2) Å], yet no *trans* effect is observed as the two Cu—-Cl bond lengths are very close [2.2344 (7) and 2.2380 (7) Å]. These bond lengths are similar to those observed for a related complex, *viz*. di­chloro-(4-{2-[4-(pyridin-2-yl)-1*H*-1,2,3-triazol-1-yl]eth­yl}morpholine)­copper(II) (Jones *et al.*, 2012 ▸).

## Supra­molecular features   

**Figure 2:**
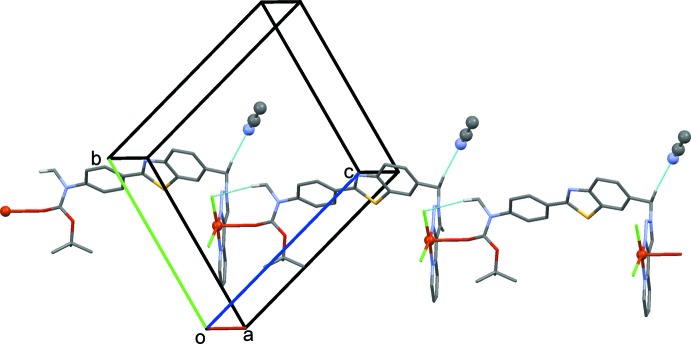
A view along the *a* axis of the aceto­nitrile solvent mol­ecules (ball and stick) linked to the polymer chains, that propagate along direction [11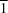], *via* a C—H⋯N hydrogen bond (see Table 2 ▸ for details). Other H atoms have been omitted for clarity.

**Figure 3:**
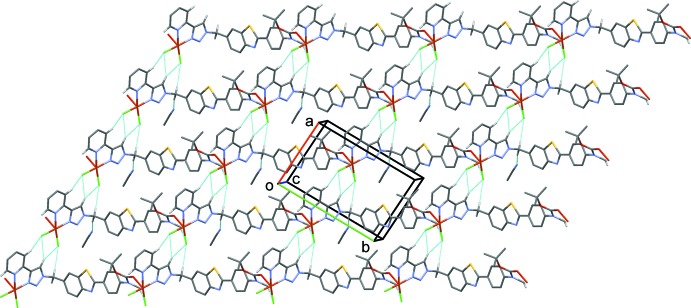
A view along the *c* axis of the crystal packing of the title compound, showing the hydrogen bonds (dashed lines; see Table 2 ▸ for details) forming sheets parallel to (011). H atoms not involved in these inter­actions have been omitted.

**Figure 4:**
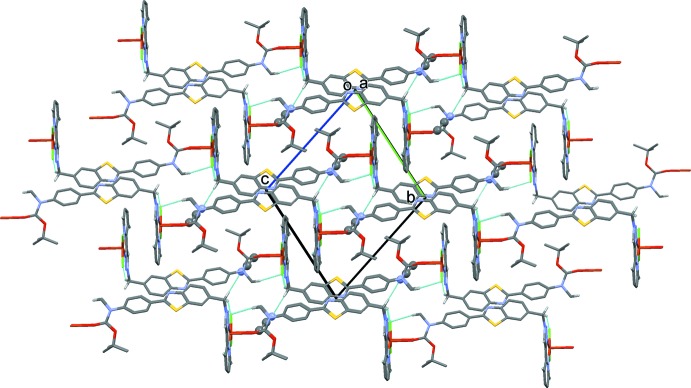
A view along the *a* axis of the crystal packing of the title compound, showing the hydrogen bonds as dashed lines (see Table 2 ▸ for details). H atoms not involved in these inter­actions have been omitted.

In the crystal, the polymer chains propagate in the [11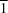] direction (Fig. 2 ▸). They are linked by C—H⋯Cl hydrogen bonds, forming sheets parallel to (011); see Fig. 3 ▸ and Table 2 ▸. The aceto­nitrile solvent mol­ecules are linked to the polymer chains within the network by C—H⋯N hydrogen bonds (Figs. 2 ▸ and 3 ▸; Table 2 ▸). The crystal packing is further consolidated by C—H⋯π inter­actions (Table 2 ▸) and offset π–π stacking inter­actions, forming a three-dimensional supra­molecular structure (Fig. 4 ▸). The offset π–π inter­actions involve inversion-related triazole and pyridine rings with inter­planar distances of 3.3848 (11) and 3.300 (1) Å [*Cg*3⋯*Cg*4^i^ = 3.6805 (15) Å, α = 4.07 (14)°, slippages are 1.63 and 1.45 Å; *Cg*3 and *Cg*4 are the centroids of rings N17–N19/C20/C21 and N27/C22–C26, respectively; symmetry code: (i) −*x*, −*y* − 1, −*z* + 2].

## Database survey   

A search of the Cambridge Structural Database (CSD, Version 5.38, update May 2017; Groom *et al.*, 2016 ▸) for pyridine-triazole copper(II) dichloride complexes gave seven hits. Two of these compounds have a similar geometry involving the copper(II) atom, *viz*. di­chloro-(4-{2-[4-(pyridin-2-yl)-1*H*-1,2,3-triazol-1-yl]eth­yl}morpholine)­copper(II) (CSD refcode MEHHEO; Jones *et al.*, 2012 ▸) and bis­(μ-chloro)­dichloro-bis­(2-{[4-(pyridin-2-yl)-1*H*-1,2,3-triazol-1-yl]meth­yl}benzo­nitrile)di-copper (UMIYEW; Bai *et al.*, 2016 ▸). As in the title compound (see Table 1 ▸), the Cu^II^ ions have fivefold coordin­ation spheres with a square-pyramidal geometry. In addition, the Cu—N_pyridine_ bond lengths [2.063 (3) and 2.075 (2) Å, respectively] are slightly longer than the Cu—N_triazole_ bond lengths [2.024 (3) and 2.005 (3) Å, respectively], while the Cu—Cl bonds lengths are very similar in both complexes [2.265 (1) and 2.242 (1) Å in MEHHEO, and 2.246 (1) and 2.264 (1) Å in UMIYEW]. However, both of these compounds are binuclear complexes, possessing inversion symmetry, with bis­(μ-chloro) Cl^−^ anions bridging the metal ions.

## Synthesis and crystallization   

The synthesis of the ligand, *tert*-butyl meth­yl[4-(6-{[4-(pyridin-2-yl)-1*H*-1,2,3-triazol-1-yl]meth­yl}benzo[*d*]thia­zol-2-yl)phen­yl]carbamate (**L**), was performed according to literature precedents (Noel *et al.*, 2013 ▸; Jones *et al.*, 2012 ▸). A mixture of 15 mg of **L** dissolved in 1 ml of aceto­nitrile, and 1.1 equiv. of CuCl_2_ dissolved in 10 ml of a mixture aceto­nitrile/H_2_O (6/3) was heated to 353 K. The mixture was cooled at room temperature, allowing a precipitate to form. The supernatant was removed and the precipitate was dissolved with a minimum volume of hot aceto­nitrile, filtered and left at room temperature in a closed vessel producing overnight pale-green plate-like crystals.
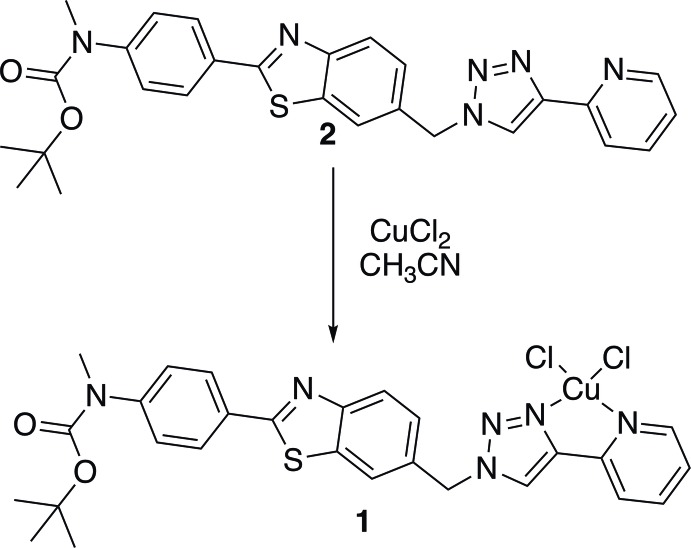


## Refinement   

Crystal data, data collection and structure refinement details are summarized in Table 3 ▸. The H atoms were all located in difference-Fourier maps, but those attached to carbon atoms were repositioned geometrically. The H atoms were initially refined with soft restraints on the bond lengths and angles to regularize their geometry [C—H = 0.93–0.98 Å with *U*_iso_(H) = 1.5*U*_eq_(C-meth­yl) and 1.2*U*_eq_(C) for other H atoms], after which the positions were refined with riding constraints (Cooper *et al.*, 2010 ▸).

## Supplementary Material

Crystal structure: contains datablock(s) I, Global. DOI: 10.1107/S2056989018000488/su5417sup1.cif

Structure factors: contains datablock(s) I. DOI: 10.1107/S2056989018000488/su5417Isup2.hkl

CCDC reference: 1815501

Additional supporting information:  crystallographic information; 3D view; checkCIF report

## Figures and Tables

**Table 1:** Selected geometric parameters (Å, °)

Cu1—O31^i^	2.508 (2)	Cu1—Cl1	2.2344 (7)
Cu1—N19	2.004 (2)	Cu1—Cl2	2.2380 (7)
Cu1—N27	2.054 (2)		
			
Cl1—Cu1—N19	168.01 (7)	Cl2—Cu1—N27	172.70 (6)

Symmetry code: (i) 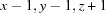.

**Table 2:** Hydrogen-bond geometry (Å, °) *Cg* is the centroid of the C4–C9 ring.

*D*—H⋯*A*	*D*—H	H⋯*A*	*D*⋯*A*	*D*—H⋯*A*
C16—H162⋯N39^ii^	0.97	2.52	3.451 (6)	161
C16—H161⋯Cl2^iii^	0.97	2.72	3.606 (3)	152
C21—H211⋯Cl1^iii^	0.94	2.81	3.633 (3)	147
C23—H231⋯Cl1^iii^	0.94	2.62	3.494 (3)	155
C26—H261⋯Cl1	0.94	2.55	3.154 (3)	122
C29—H291⋯Cl2^iv^	0.95	2.80	3.741 (3)	172
C25—H251⋯*Cg* ^v^	0.94	2.85	3.583 (3)	135

Symmetry codes: (ii) 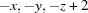; (iii) 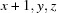; (iv) 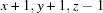; (v) 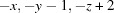.

**Table 3:** Experimental details

Crystal data	
Chemical formula	[CuCl_2_(C_27_H_26_N_6_O_2_S)]·CH_3_CN
*M*_r_	674.11
Crystal system, space group	Triclinic, *P* 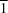
Temperature (K)	100
*a*, *b*, *c* (Å)	8.6374 (7), 13.1553 (10), 14.2243 (11)
α, β, γ (°)	73.755 (3), 73.863 (3), 84.226 (3)
*V* (Å^3^)	1490.1 (2)
*Z*	2
Radiation type	Mo *K*α
μ (mm^−1^)	1.02
Crystal size (mm)	0.12 × 0.09 × 0.02

Data collection	
Diffractometer	Bruker Kappa APEXII
Absorption correction	Multi-scan (*SADABS*; Bruker, 2006 ▸)
*T*_min_, *T*_max_	0.91, 0.98
No. of measured, independent and observed [*I* > 2.0σ(*I*)] reflections	26982, 5475, 4358
*R*_int_	0.053
(sin θ/λ)_max_ (Å^−1^)	0.603

Refinement	
*R*[*F* ^2^ > 2σ(*F* ^2^)], *wR*(*F* ^2^), *S*	0.037, 0.036, 1.05
No. of reflections	4062
No. of parameters	379
H-atom treatment	H-atom parameters constrained
Δρ_max_, Δρ_min_ (e Å^−3^)	0.45, −0.36

Computer programs: *APEX2* and *SAINT* (Bruker, 2006 ▸), *SUPERFLIP* (Palatinus & Chapuis, 2007 ▸), *Mercury* (Macrae *et al.*, 2008 ▸), *CRYSTALS* (Betteridge *et al.*, 2003 ▸) and *PLATON* (Spek, 2009 ▸). Weighting scheme: Chebychev polynomial (Watkin, 1994 ▸; Prince, 1982 ▸)

## supplementary crystallographic information

### Crystal data

**Table:**

[CuCl_2_(C_27_H_26_N_6_O_2_S)]·CH_3_CN	*Z* = 2
*M**_r_* = 674.11	*F*(000) = 694
Triclinic, *P*1	*D*_x_ = 1.502 Mg m^−^^3^
Hall symbol: -P 1	Mo *K*α radiation, λ = 0.71073 Å
*a* = 8.6374 (7) Å	Cell parameters from 7700 reflections
*b* = 13.1553 (10) Å	θ = 2–25°
*c* = 14.2243 (11) Å	µ = 1.02 mm^−^^1^
α = 73.755 (3)°	*T* = 100 K
β = 73.863 (3)°	Plate, pale green
γ = 84.226 (3)°	0.12 × 0.09 × 0.02 mm
*V* = 1490.1 (2) Å^3^	

### Data collection

**Table:**

Bruker Kappa APEXII diffractometer	4358 reflections with *I* > 2.0σ(*I*)
Graphite monochromator	*R*_int_ = 0.053
φ & ω scans	θ_max_ = 25.4°, θ_min_ = 1.6°
Absorption correction: multi-scan (SADABS; Bruker, 2006)	*h* = −10→8
*T*_min_ = 0.91, *T*_max_ = 0.98	*k* = −15→15
26982 measured reflections	*l* = −17→17
5475 independent reflections	

### Refinement

**Table:**

Refinement on *F*	Primary atom site location: other
Least-squares matrix: full	Secondary atom site location: difference Fourier map
*R*[*F*^2^ > 2σ(*F*^2^)] = 0.037	Hydrogen site location: difference Fourier map
*wR*(*F*^2^) = 0.036	H-atom parameters constrained
*S* = 1.05	Method, part 1, Chebychev polynomial, (Watkin, 1994; *P*rince, 1982) [*w*eight] = 1.0/[A_0_*T_0_(x) + A_1_*T_1_(x) ··· + A_n-1_]*T_n-1_(x)] where A_i_ are the Chebychev coefficients listed belo*w* and x = *F* /*F*max Method = Robust Weighting (*P*rince, 1982) W = [*w*eight] * [1-(delta*F*/6*sigma*F*)^2^]^2^ A_i_ are: 0.270 0.160 0.128
4062 reflections	(Δ/σ)_max_ = 0.001
379 parameters	Δρ_max_ = 0.45 e Å^−^^3^
0 restraints	Δρ_min_ = −0.36 e Å^−^^3^

### Special details

**Table:**

Experimental. The crystal was placed in the cold stream of an Oxford Cryosystems open-flow nitrogen cryostat (Cosier & Glazer, 1986) with a nominal stability of 0.1 K. Cosier, J. & Glazer, A.M., 1986. J. Appl. Cryst. 105-107.

### Fractional atomic coordinates and isotropic or equivalent isotropic displacement parameters (Å^2^)

**Table:**

	*x*	*y*	*z*	*U*_iso_*/*U*_eq_	
S1	0.49721 (8)	−0.10533 (5)	0.91493 (5)	0.0178	
Cu1	−0.26343 (4)	−0.55390 (3)	1.17008 (3)	0.0153	
Cl1	−0.43826 (8)	−0.62080 (6)	1.11470 (6)	0.0283	
C2	0.4057 (3)	0.0195 (2)	0.8788 (2)	0.0166	
Cl2	−0.45219 (8)	−0.45258 (6)	1.24664 (5)	0.0241	
N3	0.2719 (3)	0.03835 (18)	0.94172 (17)	0.0182	
C4	0.0978 (3)	−0.0538 (2)	1.1089 (2)	0.0198	
C5	0.0743 (3)	−0.1456 (2)	1.1862 (2)	0.0184	
C6	0.1821 (3)	−0.2326 (2)	1.18434 (19)	0.0154	
C7	0.3190 (3)	−0.2270 (2)	1.1035 (2)	0.0168	
C8	0.3417 (3)	−0.1348 (2)	1.0253 (2)	0.0157	
C9	0.2337 (3)	−0.0477 (2)	1.0261 (2)	0.0164	
C10	0.4785 (3)	0.0928 (2)	0.7807 (2)	0.0167	
C11	0.6322 (3)	0.0724 (2)	0.7241 (2)	0.0196	
C12	0.6982 (3)	0.1394 (2)	0.6295 (2)	0.0218	
C13	0.6083 (3)	0.2264 (2)	0.5891 (2)	0.0205	
C14	0.4567 (4)	0.2492 (2)	0.6463 (2)	0.0226	
C15	0.3921 (3)	0.1834 (2)	0.7420 (2)	0.0197	
C16	0.1480 (3)	−0.3355 (2)	1.2670 (2)	0.0176	
N17	0.0898 (3)	−0.41369 (17)	1.22896 (16)	0.0142	
N18	−0.0676 (3)	−0.41805 (17)	1.23796 (16)	0.0156	
N19	−0.0798 (2)	−0.48723 (16)	1.18938 (17)	0.0149	
C20	0.0688 (3)	−0.5257 (2)	1.14907 (19)	0.0143	
C21	0.1801 (3)	−0.4783 (2)	1.17465 (19)	0.0165	
C22	0.0753 (3)	−0.6034 (2)	1.09231 (19)	0.0153	
C23	0.2169 (3)	−0.6451 (2)	1.0407 (2)	0.0177	
C24	0.2065 (3)	−0.7194 (2)	0.9903 (2)	0.0206	
C25	0.0551 (3)	−0.7488 (2)	0.9927 (2)	0.0191	
C26	−0.0798 (3)	−0.7017 (2)	1.0431 (2)	0.0171	
N27	−0.0723 (3)	−0.63013 (17)	1.09261 (16)	0.0145	
N28	0.6716 (3)	0.29292 (18)	0.48943 (17)	0.0230	
C29	0.6889 (4)	0.4067 (2)	0.4767 (2)	0.0320	
C30	0.7287 (4)	0.2548 (2)	0.4066 (2)	0.0228	
O31	0.7877 (3)	0.30981 (16)	0.32214 (15)	0.0289	
O32	0.7071 (3)	0.15044 (16)	0.43036 (15)	0.0304	
C33	0.7874 (4)	0.0873 (2)	0.3586 (2)	0.0286	
C34	0.7093 (4)	0.1108 (3)	0.2724 (2)	0.0303	
C35	0.9671 (4)	0.1073 (3)	0.3221 (3)	0.0478	
C36	0.7533 (6)	−0.0256 (3)	0.4247 (3)	0.0546	
C37	0.3253 (6)	0.2890 (4)	0.3651 (4)	0.0763	
C38	0.1812 (5)	0.2908 (3)	0.4432 (3)	0.0502	
N39	0.0648 (6)	0.2875 (4)	0.5048 (4)	0.0862	
H41	0.0246	0.0045	1.1119	0.0245*	
H51	−0.0172	−0.1504	1.2437	0.0226*	
H71	0.3946	−0.2857	1.1026	0.0214*	
H111	0.6940	0.0122	0.7511	0.0246*	
H121	0.8046	0.1270	0.5925	0.0270*	
H141	0.3970	0.3095	0.6193	0.0274*	
H151	0.2892	0.2001	0.7807	0.0247*	
H161	0.2462	−0.3640	1.2865	0.0221*	
H162	0.0655	−0.3234	1.3253	0.0219*	
H211	0.2925	−0.4869	1.1601	0.0196*	
H231	0.3174	−0.6224	1.0400	0.0224*	
H241	0.2997	−0.7503	0.9546	0.0264*	
H251	0.0439	−0.7998	0.9599	0.0235*	
H261	−0.1829	−0.7191	1.0427	0.0217*	
H291	0.6493	0.4483	0.4217	0.0499*	
H292	0.6300	0.4275	0.5370	0.0497*	
H293	0.8010	0.4230	0.4655	0.0508*	
H341	0.7619	0.0674	0.2266	0.0456*	
H342	0.5970	0.0936	0.2989	0.0468*	
H343	0.7196	0.1844	0.2362	0.0455*	
H351	1.0195	0.0571	0.2860	0.0706*	
H352	1.0120	0.0978	0.3794	0.0711*	
H353	0.9903	0.1784	0.2784	0.0709*	
H361	0.7925	−0.0372	0.4838	0.0841*	
H362	0.8064	−0.0739	0.3855	0.0839*	
H363	0.6376	−0.0356	0.4445	0.0840*	
H371	0.3941	0.2312	0.3908	0.1152*	
H372	0.3774	0.3554	0.3457	0.1152*	
H373	0.2956	0.2769	0.3077	0.1154*	

### Atomic displacement parameters (Å^2^)

**Table:**

	*U*^11^	*U*^22^	*U*^33^	*U*^12^	*U*^13^	*U*^23^
S1	0.0155 (3)	0.0172 (3)	0.0189 (3)	0.0004 (2)	−0.0021 (3)	−0.0046 (3)
Cu1	0.01124 (16)	0.01627 (17)	0.02098 (18)	−0.00100 (12)	−0.00448 (12)	−0.00854 (13)
Cl1	0.0169 (3)	0.0348 (4)	0.0440 (4)	0.0014 (3)	−0.0124 (3)	−0.0236 (3)
C2	0.0181 (13)	0.0180 (13)	0.0193 (13)	−0.0008 (10)	−0.0095 (11)	−0.0091 (11)
Cl2	0.0161 (3)	0.0283 (4)	0.0327 (4)	0.0033 (3)	−0.0062 (3)	−0.0174 (3)
N3	0.0194 (11)	0.0178 (11)	0.0196 (12)	0.0015 (9)	−0.0055 (9)	−0.0086 (10)
C4	0.0200 (13)	0.0200 (14)	0.0228 (14)	0.0015 (11)	−0.0054 (11)	−0.0121 (12)
C5	0.0191 (13)	0.0198 (14)	0.0191 (13)	−0.0021 (10)	−0.0042 (11)	−0.0098 (11)
C6	0.0170 (12)	0.0169 (13)	0.0166 (13)	−0.0047 (10)	−0.0069 (10)	−0.0073 (11)
C7	0.0160 (12)	0.0177 (13)	0.0206 (14)	−0.0011 (10)	−0.0064 (11)	−0.0094 (11)
C8	0.0130 (12)	0.0187 (13)	0.0191 (13)	−0.0032 (10)	−0.0049 (10)	−0.0090 (11)
C9	0.0169 (12)	0.0175 (13)	0.0187 (13)	−0.0023 (10)	−0.0063 (10)	−0.0087 (11)
C10	0.0201 (13)	0.0158 (13)	0.0184 (13)	−0.0029 (10)	−0.0073 (11)	−0.0077 (11)
C11	0.0207 (13)	0.0217 (14)	0.0188 (14)	0.0003 (11)	−0.0075 (11)	−0.0071 (11)
C12	0.0218 (14)	0.0259 (15)	0.0192 (14)	−0.0016 (11)	−0.0054 (11)	−0.0082 (12)
C13	0.0286 (15)	0.0157 (13)	0.0188 (14)	−0.0040 (11)	−0.0058 (11)	−0.0064 (11)
C14	0.0311 (15)	0.0185 (14)	0.0210 (14)	0.0044 (12)	−0.0105 (12)	−0.0081 (12)
C15	0.0224 (14)	0.0198 (14)	0.0192 (14)	0.0009 (11)	−0.0042 (11)	−0.0106 (11)
C16	0.0187 (13)	0.0189 (13)	0.0182 (13)	−0.0012 (10)	−0.0053 (11)	−0.0088 (11)
N17	0.0147 (10)	0.0149 (11)	0.0140 (11)	−0.0031 (8)	−0.0031 (8)	−0.0050 (9)
N18	0.0148 (10)	0.0149 (11)	0.0172 (11)	−0.0030 (8)	−0.0029 (9)	−0.0046 (9)
N19	0.0137 (10)	0.0113 (10)	0.0194 (11)	−0.0010 (8)	−0.0043 (9)	−0.0031 (9)
C20	0.0131 (12)	0.0139 (12)	0.0147 (12)	−0.0010 (10)	−0.0024 (10)	−0.0029 (10)
C21	0.0166 (12)	0.0180 (13)	0.0161 (13)	−0.0004 (10)	−0.0037 (10)	−0.0072 (11)
C22	0.0163 (12)	0.0146 (13)	0.0159 (13)	−0.0012 (10)	−0.0074 (10)	−0.0019 (10)
C23	0.0154 (12)	0.0202 (13)	0.0205 (14)	0.0019 (10)	−0.0069 (11)	−0.0087 (11)
C24	0.0219 (14)	0.0192 (14)	0.0215 (14)	0.0025 (11)	−0.0064 (11)	−0.0068 (11)
C25	0.0266 (14)	0.0140 (13)	0.0187 (14)	0.0005 (11)	−0.0074 (11)	−0.0062 (11)
C26	0.0202 (13)	0.0152 (12)	0.0176 (13)	−0.0030 (10)	−0.0069 (11)	−0.0044 (11)
N27	0.0169 (11)	0.0148 (11)	0.0125 (11)	−0.0016 (9)	−0.0057 (9)	−0.0025 (9)
N28	0.0349 (14)	0.0170 (12)	0.0162 (12)	−0.0019 (10)	−0.0036 (10)	−0.0052 (10)
C29	0.053 (2)	0.0167 (14)	0.0221 (15)	−0.0005 (14)	−0.0055 (14)	−0.0035 (12)
C30	0.0293 (15)	0.0183 (14)	0.0213 (15)	−0.0010 (12)	−0.0069 (12)	−0.0057 (12)
O31	0.0433 (13)	0.0218 (11)	0.0165 (10)	−0.0017 (9)	−0.0011 (9)	−0.0033 (9)
O32	0.0522 (14)	0.0183 (10)	0.0181 (10)	−0.0036 (9)	−0.0013 (9)	−0.0078 (8)
C33	0.0434 (18)	0.0222 (15)	0.0241 (16)	0.0037 (13)	−0.0105 (14)	−0.0125 (13)
C34	0.0380 (17)	0.0315 (17)	0.0265 (16)	−0.0045 (14)	−0.0108 (14)	−0.0125 (13)
C35	0.040 (2)	0.054 (2)	0.068 (3)	0.0172 (17)	−0.0253 (19)	−0.042 (2)
C36	0.110 (4)	0.0202 (17)	0.037 (2)	0.0028 (19)	−0.023 (2)	−0.0102 (15)
C37	0.051 (3)	0.056 (3)	0.084 (4)	−0.005 (2)	0.021 (2)	0.004 (3)
C38	0.038 (2)	0.058 (3)	0.049 (2)	0.0078 (18)	−0.0059 (19)	−0.015 (2)
N39	0.073 (3)	0.099 (4)	0.072 (3)	0.013 (3)	0.005 (2)	−0.029 (3)

### Geometric parameters (Å, º)

**Table:**

S1—C2	1.754 (3)	C20—C21	1.373 (4)
S1—C8	1.736 (3)	C20—C22	1.458 (4)
Cu1—O31^i^	2.508 (2)	C21—H211	0.937
Cu1—N19	2.004 (2)	C22—C23	1.388 (4)
Cu1—N27	2.054 (2)	C22—N27	1.355 (3)
Cu1—Cl1	2.2344 (7)	C23—C24	1.386 (4)
Cu1—Cl2	2.2380 (7)	C23—H231	0.943
C2—N3	1.300 (3)	C24—C25	1.390 (4)
C2—C10	1.468 (4)	C24—H241	0.946
N3—C9	1.387 (4)	C25—C26	1.377 (4)
C4—C5	1.375 (4)	C25—H251	0.943
C4—C9	1.403 (4)	C26—N27	1.339 (3)
C4—H41	0.947	C26—H261	0.944
C5—C6	1.402 (4)	N28—C29	1.474 (4)
C5—H51	0.959	N28—C30	1.356 (4)
C6—C7	1.393 (4)	C29—H291	0.949
C6—C16	1.516 (4)	C29—H292	0.964
C7—C8	1.385 (4)	C29—H293	0.973
C7—H71	0.961	C30—O31	1.214 (3)
C8—C9	1.403 (4)	C30—O32	1.338 (3)
C10—C11	1.392 (4)	O32—C33	1.476 (3)
C10—C15	1.401 (4)	C33—C34	1.503 (4)
C11—C12	1.386 (4)	C33—C35	1.518 (5)
C11—H111	0.959	C33—C36	1.526 (5)
C12—C13	1.395 (4)	C34—H341	0.974
C12—H121	0.948	C34—H342	0.963
C13—C14	1.390 (4)	C34—H343	0.962
C13—N28	1.431 (4)	C35—H351	0.952
C14—C15	1.389 (4)	C35—H352	0.969
C14—H141	0.948	C35—H353	0.972
C15—H151	0.948	C36—H361	0.960
C16—N17	1.473 (3)	C36—H362	0.961
C16—H161	0.973	C36—H363	0.972
C16—H162	0.971	C37—C38	1.422 (6)
N17—N18	1.336 (3)	C37—H371	0.970
N17—C21	1.352 (3)	C37—H372	0.957
N18—N19	1.315 (3)	C37—H373	0.977
N19—C20	1.362 (3)	C38—N39	1.131 (6)
			
C2—S1—C8	89.01 (13)	C20—C21—N17	103.8 (2)
O31^i^—Cu1—Cl1	107.68 (6)	C20—C21—H211	130.3
O31^i^—Cu1—Cl2	100.13 (5)	N17—C21—H211	125.8
Cl1—Cu1—Cl2	93.31 (3)	C20—C22—C23	124.4 (2)
O31^i^—Cu1—N19	80.21 (8)	C20—C22—N27	113.2 (2)
Cl1—Cu1—N19	168.01 (7)	C23—C22—N27	122.4 (2)
Cl2—Cu1—N19	94.14 (6)	C22—C23—C24	118.6 (2)
O31^i^—Cu1—N27	83.26 (8)	C22—C23—H231	119.9
Cl1—Cu1—N27	91.79 (6)	C24—C23—H231	121.5
Cl2—Cu1—N27	172.70 (6)	C23—C24—C25	119.0 (2)
N19—Cu1—N27	80.00 (8)	C23—C24—H241	121.6
S1—C2—N3	115.9 (2)	C25—C24—H241	119.5
S1—C2—C10	119.49 (19)	C24—C25—C26	118.9 (2)
N3—C2—C10	124.6 (2)	C24—C25—H251	121.0
C2—N3—C9	110.5 (2)	C26—C25—H251	120.0
C5—C4—C9	118.8 (3)	C25—C26—N27	123.0 (2)
C5—C4—H41	120.6	C25—C26—H261	119.4
C9—C4—H41	120.6	N27—C26—H261	117.6
C4—C5—C6	121.8 (3)	C22—N27—C26	118.0 (2)
C4—C5—H51	119.6	C22—N27—Cu1	115.25 (17)
C6—C5—H51	118.6	C26—N27—Cu1	126.77 (18)
C5—C6—C7	120.1 (2)	C13—N28—C29	118.9 (2)
C5—C6—C16	120.9 (2)	C13—N28—C30	122.9 (2)
C7—C6—C16	119.0 (2)	C29—N28—C30	118.0 (2)
C6—C7—C8	118.0 (2)	N28—C29—H291	110.7
C6—C7—H71	120.5	N28—C29—H292	111.1
C8—C7—H71	121.5	H291—C29—H292	108.1
S1—C8—C7	128.5 (2)	N28—C29—H293	110.9
S1—C8—C9	109.2 (2)	H291—C29—H293	109.6
C7—C8—C9	122.3 (2)	H292—C29—H293	106.3
C8—C9—C4	119.0 (2)	N28—C30—O31	123.7 (3)
C8—C9—N3	115.5 (2)	N28—C30—O32	111.1 (2)
C4—C9—N3	125.5 (2)	O31—C30—O32	125.3 (3)
C2—C10—C11	120.7 (2)	Cu1^ii^—O31—C30	146.2 (2)
C2—C10—C15	120.1 (2)	C30—O32—C33	121.0 (2)
C11—C10—C15	119.2 (2)	O32—C33—C34	109.9 (3)
C10—C11—C12	120.6 (3)	O32—C33—C35	110.4 (2)
C10—C11—H111	119.8	C34—C33—C35	112.3 (3)
C12—C11—H111	119.5	O32—C33—C36	101.8 (2)
C11—C12—C13	119.9 (3)	C34—C33—C36	110.6 (3)
C11—C12—H121	120.5	C35—C33—C36	111.4 (3)
C13—C12—H121	119.7	C33—C34—H341	109.0
C12—C13—C14	119.8 (3)	C33—C34—H342	109.2
C12—C13—N28	120.4 (2)	H341—C34—H342	109.1
C14—C13—N28	119.7 (3)	C33—C34—H343	110.2
C13—C14—C15	120.2 (3)	H341—C34—H343	109.5
C13—C14—H141	119.7	H342—C34—H343	109.8
C15—C14—H141	120.1	C33—C35—H351	109.2
C10—C15—C14	120.1 (3)	C33—C35—H352	110.3
C10—C15—H151	120.3	H351—C35—H352	107.5
C14—C15—H151	119.6	C33—C35—H353	112.0
C6—C16—N17	109.6 (2)	H351—C35—H353	109.2
C6—C16—H161	110.1	H352—C35—H353	108.5
N17—C16—H161	108.3	C33—C36—H361	110.0
C6—C16—H162	109.9	C33—C36—H362	108.5
N17—C16—H162	108.7	H361—C36—H362	110.0
H161—C16—H162	110.3	C33—C36—H363	108.6
C16—N17—N18	119.7 (2)	H361—C36—H363	110.1
C16—N17—C21	127.2 (2)	H362—C36—H363	109.6
N18—N17—C21	112.7 (2)	C38—C37—H371	108.2
N17—N18—N19	105.46 (19)	C38—C37—H372	109.3
N18—N19—Cu1	134.95 (17)	H371—C37—H372	110.9
N18—N19—C20	110.4 (2)	C38—C37—H373	107.7
Cu1—N19—C20	114.51 (16)	H371—C37—H373	109.9
N19—C20—C21	107.6 (2)	H372—C37—H373	110.6
N19—C20—C22	116.9 (2)	C37—C38—N39	176.6 (5)
C21—C20—C22	135.4 (2)		

Symmetry codes: (i) *x*−1, *y*−1, *z*+1; (ii) *x*+1, *y*+1, *z*−1.

### Hydrogen-bond geometry (Å, º)

Cg is the centroid of the C4–C9 ring.

**Table:**

*D*—H···*A*	*D*—H	H···*A*	*D*···*A*	*D*—H···*A*
C16—H162···N39^iii^	0.97	2.52	3.451 (6)	161
C16—H161···Cl2^iv^	0.97	2.72	3.606 (3)	152
C21—H211···Cl1^iv^	0.94	2.81	3.633 (3)	147
C23—H231···Cl1^iv^	0.94	2.62	3.494 (3)	155
C26—H261···Cl1	0.94	2.55	3.154 (3)	122
C29—H291···Cl2^ii^	0.95	2.80	3.741 (3)	172
C25—H251···*Cg*^v^	0.94	2.85	3.583 (3)	135

Symmetry codes: (ii) *x*+1, *y*+1, *z*−1; (iii) −*x*, −*y*, −*z*+2; (iv) *x*+1, *y*, *z*; (v) −*x*, −*y*−1, −*z*+2.
